# Coevolution of Interacting Fertilization Proteins

**DOI:** 10.1371/journal.pgen.1000570

**Published:** 2009-07-24

**Authors:** Nathaniel L. Clark, Joe Gasper, Masashi Sekino, Stevan A. Springer, Charles F. Aquadro, Willie J. Swanson

**Affiliations:** 1 Department of Molecular Biology and Genetics, Cornell University, Ithaca, New York, United States of America; 2 Department of Genome Sciences, University of Washington, Seattle, Washington, United States of America; 3 Tohoku National Fisheries Research Institute, Fisheries Research Agency, Shiogama, Miyagi, Japan; University of Aarhus, Denmark

## Abstract

Reproductive proteins are among the fastest evolving in the proteome, often due to the consequences of positive selection, and their rapid evolution is frequently attributed to a coevolutionary process between interacting female and male proteins. Such a process could leave characteristic signatures at coevolving genes. One signature of coevolution, predicted by sexual selection theory, is an association of alleles between the two genes. Another predicted signature is a correlation of evolutionary rates during divergence due to compensatory evolution. We studied female–male coevolution in the abalone by resequencing sperm lysin and its interacting egg coat protein, VERL, in populations of two species. As predicted, we found intergenic linkage disequilibrium between lysin and VERL, despite our demonstration that they are not physically linked. This finding supports a central prediction of sexual selection using actual genotypes, that of an association between a male trait and its female preference locus. We also created a novel likelihood method to show that lysin and VERL have experienced correlated rates of evolution. These two signatures of coevolution can provide statistical rigor to hypotheses of coevolution and could be exploited for identifying coevolving proteins *a priori*. We also present polymorphism-based evidence for positive selection and implicate recent selective events at the specific structural regions of lysin and VERL responsible for their species-specific interaction. Finally, we observed deep subdivision between VERL alleles in one species, which matches a theoretical prediction of sexual conflict. Thus, abalone fertilization proteins illustrate how coevolution can lead to reproductive barriers and potentially drive speciation.

## Author Summary

When a sperm meets an egg, it must display the correct recognition proteins to achieve fertilization. Given the importance of fertilization one would think these proteins are perfected and do not change over time; however, recent studies show that they do change and quite rapidly. Thus, the sperm and egg must change together in harmony, through a process called coevolution, so the species can successfully reproduce. We followed the sperm–egg coevolutionary process at the level of genes: one that makes the protective egg coat and a sperm gene which opens that coat for fertilization. By examining their DNA sequences in two abalone species, we revealed two coevolutionary signatures. In one case, we discovered an association of variants between the egg and sperm genes, the origin of which could be strong preference for compatible variants. In the second case, we demonstrated that both genes changed at correlated rates over millions of years of evolution. Whenever one gene had accelerated in one species, the other showed a parallel acceleration in that same species. These unique signatures help us to understand coevolution by revealing its strength within natural populations and by showing that it has acted consistently over long time periods.

## Introduction

Given the importance of reproduction for evolutionary fitness, one might predict that reproductive proteins would be highly conserved, especially those required for interaction between sperm and egg. The requirement of molecular recognition between interacting proteins should result in sequence conservation, and indeed, there is proteome-wide evidence that interaction interfaces are more highly conserved than other surface residues [1]. Yet many key reproductive proteins evolve rapidly and are driven to do so by positive selection [2]. Furthermore, several fertilization proteins are driven to change at the specific regions mediating sperm-egg recognition. This observation stands in stark contrast to the general conservation of interaction interfaces. The driving force behind the initial divergence of fertilization proteins is unknown and is a subject of active investigation. Some proposed hypotheses are sexual selection, the creation of reproductive barriers between hybridizing populations (reinforcement), and a sexual conflict over the fertilization rate [2]. Regardless of which force drives their initial divergence, the interaction between sperm and egg should be maintained through coevolution, in which one or both proteins adaptively compensate for changes in the other. Such female-male coevolution has often been proposed to contribute to the rapid evolution of reproductive proteins [3]–[6], but specific tests of this hypothesis have not been performed.

The process of coevolution could leave characteristic signatures on interacting fertilization genes. In populations with multiple alleles, sperm-egg pairs expressing compatible alleles would have fertilization rates exceeding those of relatively incompatible pairs, especially in the presence of sperm competition. This difference in rates would create an association of compatible alleles within zygotes. Hence, one predicted signature, apparent in populations, is a genetic association of alleles between the two interacting genes. Such an association could be observed as intergenic linkage disequilibrium (LD). This prediction has also been made in terms of sexual selection, in which a genetic association develops between a male trait and female preference for that trait [7],[8].

Another potential signature of coevolution is found in gene phylogenies in which coevolution could create a long-term correlation of evolutionary rates between the two genes [9],[10]. During coevolution, acceleration in the rate of change of one protein would cause its cognate to compensate in order to maintain their interaction. Hence, rates of evolution would correlate between corresponding branches of their phylogenetic trees. This prediction has been supported by multiple studies which found correlations of branch lengths between interacting proteins [9]–[14]. Such a signature could also be used to test for long-term coevolution between interacting fertilization proteins.

Interacting sperm-egg protein pairs are currently known in only two taxonomic groups, abalone and sea urchins [15],[16]. Abalone are marine mollusks that release eggs and sperm into the water column so that recognition proteins on gamete surfaces are largely responsible for species-specificity. Abalone fertilization is partially controlled by the proteins lysin and VERL (vitelline envelope receptor for lysin). The large glycoprotein VERL (3,761 amino acids) contains 22 tandem repeat units which are thought to bind each other to create structural fibers of the egg vitelline envelope, a major barrier to sperm [15]. Sperm release lysin (∼150 amino acids) onto the vitelline envelope where it non-enzymatically unravels VERL fibers, opening a hole for sperm entry [17]. It is hypothesized that lysin disrupts the self-interaction of VERL repeat units by competing for hydrogen bonds [15]. The interaction between vitelline envelopes and lysin is highly species-specific, contributing to reproductive isolation between species [15],[18]. For example, fertilization efficiency is low in a hybrid cross of *Haliotis rufescens* eggs and *H. fulgens* sperm (∼10%). Yet by adding purified *H. rufescens* lysin, fertilization by *H. fulgens* sperm reaches 90%, significantly rescuing the efficiency of heterospecific sperm [19]. This biochemical rescue experiment is analogous to genetic rescue experiments and demonstrates the species-specificity of the lysin-egg interaction.

Lysin and VERL show robust signs of positive selection in their divergence between species. Likelihood methods infer both of them to have a class of codons experiencing a significant excess of amino acid substitutions compared to silent substitutions [20],[21]. Positive selection on lysin was mapped to codons in the structural regions that are important for species-specific dissolution of vitelline envelopes [18],[22]. Positive selection on VERL was found at repeats 1 and 2, but not in the remainder of its repeat array [21]. It has been proposed that repeats 1 and 2 are responsible for the species-specificity of the lysin-VERL interaction because the first 2 repeats evolve independently, having diverged from the rest of the array and from each other [23]. The remaining repeats 3 through 22 are highly homogenized by gene conversion or unequal crossing-over and share greater than 95% nucleotide identity. In summary, divergence at lysin and VERL indicates recurrent bouts of positive selection driving amino acid substitutions at the specific structural regions responsible for their interaction. This observation suggests the presence of a coevolutionary process to maintain species-specific sperm-egg recognition.

We used the well-characterized interaction between lysin and VERL to test predictions about adaptive coevolution and found support for both hypothesized signatures. We also present polymorphism-based evidence that positive selection acts on VERL and lysin, providing independent support to conclusions from divergence studies. Finally, we discovered an agreement between observed allele frequencies at these genes and predictions made by mathematical models of sexual conflict. Overall, studying both the female and male proteins revealed a more complete picture of reproductive protein evolution and allowed us to address general questions about coevolution.

## Results

### Species, Populations, and Genes of Study

To examine intraspecific patterns of fertilization protein evolution, we sequenced lysin, VERL, and non-reproductive genes in populations of two abalone species: *Haliotis corrugata*, the pink abalone, and *H. fulgens*, the green abalone. For brevity, we will refer to each species by its common name, pink or green. These two species are distributed along the Pacific coast of North America from Point Conception, California, USA to Baja California, Mexico [24]. Population samples of 28 pink and 16 green individuals were collected via non-destructive tentacle clips off Point Loma, San Diego, California. In addition to lysin and VERL, we sequenced genes with no known reproductive function to estimate baseline levels of polymorphism in these populations and to control for population demographic effects on tests of neutrality. Mitochondrial *cytochrome oxidase I* (*COI*) was sequenced in all individuals of both species. Three additional non-reproductive loci were sequenced in pink abalone. These loci were introns of three nuclear genes encoding slowly evolving, non-reproductive proteins (*rib*, *eef*, and *rtp*: see Methods). Two non-reproductive loci were sequenced in green abalone. One was the intron of a nuclear gene (*rib*), and the other included intron and exon sequence from the gene encoding cellulase (*cel*: see Methods).

### Polymorphism at VERL and Lysin

VERL consists of a non-repetitive N-terminal region, an array of 22 large repeats (each ∼153 amino acids), and a non-repetitive C-terminal region [23]. VERL is too repetitive to sequence in its entirety, so we analyzed the regions accessible by PCR: the first two repeats, the last repeat (representing the 20 repeats which are homogenized by concerted evolution), and the unique C-terminal sequence, each of which was sequenced in individuals of both populations.

**Figure 1:**
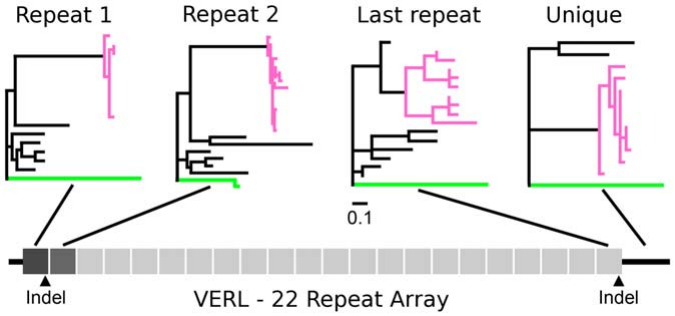
Variation in polymorphism between VERL repeat units and species. Phylogenies of each segment of VERL sequenced in this study: repeat 1, repeat 2, the last repeat, and the unique C-terminal region. Pink and green branches represent alleles from the pink and green abalone species. Other species and internal branches are shown in black. The repeat unit phylogenies contain a total of 8 species, and the ‘unique’ region has 4 species. While the pink population contains several alleles, the green population contains only two. Repeat unit types 1, 2, and 3–22 evolve independently. Repeats 3–22 evolve by concerted evolution and are represented by the last repeat. Note the deep divergence in the last pink repeat unit relative to other regions of VERL. Black arrowheads indicate the positions of two unrelated, 11-amino acid indels in the pink population.

Patterns of intraspecific polymorphism at VERL are very different between pink and green abalone. In green abalone, VERL has only a single polymorphism in all 1,470 aligned base pairs (Table 1). Hence, polymorphism (θ_W_) in green VERL is close to zero (Table 1). The single SNP is non-synonymous and is present at a moderate frequency (derived allele 16%). In the pink abalone, nucleotide diversity at VERL is much higher and even differs significantly between gene regions (Table 2). Alleles of repeats 1 and 2 are closely related and coalesce in the recent past (Figure 1), although there are multiple coding differences between them. They contain 13 non-synonymous polymorphisms and a coding insertion-deletion (indel) positioned between repeats 1 and 2. This indel variation is at a small repeat of 11 amino acids present in 1 to 4 copies in the population. In contrast, the last repeat of VERL in pinks (representing repeats 3–22) is the most polymorphic region that we sequenced. Compared with repeats 1 and 2, there is a significant excess of polymorphism in the last repeat (Hudson-Kreitman-Aguade test (HKA), P = 0.0097) [25]. This difference is remarkable because repeats 1 and 2 directly adjoin repeats 3–22 without intervening introns. The high level of polymorphism within the last repeat is evident in its repeat unit phylogeny (Figure 1). The last repeat also contains a polymorphic indel of 11 amino acids. This indel does not show homology to the N-terminal indel mentioned above, nor is it in linkage disequilibrium with it.

**Table 1:** Green abalone polymorphism and divergence.

region	nt	cds nt	S	S_N_	θ_W_	π	K_JC_	*d*_N_/*d*_S_
lysin	2143	438	6	0	0.0007	0.0008	0.101	1.19
VERL repeats 1&2	870	870	1	1	0.0003	0.0003	0.101	0.61
VERL last repeat & C-terminus	600	600	0	0	0	0	0.071	0.83
non-reproductive loci								
nuclear *rib*	910	0	11	n/a	0.0036	n.d.	0.090	n/a
nuclear *cel*	729	0	10	n/a	0.0137	n.d.	0.072	n/a
mito. *COI*	498	498	6	0	0.0043	0.0043	0.109	0

N = 16 individuals. nt = nucleotides, cds = coding sequence, S = polymorphic sites, S_N_ = nonsynonymous polymorphic sites, θ_W_ = Watterson's theta, π = nucleotide diversity, K_JC_ = Jukes-Cantor corrected distance to outgroup, *d*_N_/*d*_S_ = nonsynonymous/synonymous divergence, n/a = not applicable, n.d. = not determined.

**Table 2:** Pink abalone polymorphism and divergence.

region	nt	cds nt	polymorphism				neutrality statistics			divergence	
			S	S_N_	θ_W_	π	D_Taj_	D_FL_	H_FW_	K_JC_	*d*_N_/*d*_S_
lysin	5899	453	36	0	0.0014	0.0011	−0.55	1.23	−5.98	0.043	3.04
VERL repeats 1&2	1195	1195	26	13	0.0049	0.0035	−0.91	−0.25	−1.05	0.097	0.61
VERL last repeat	306	306	13	8	0.0093	0.0126	1.06	1.59*	−3.42	0.028	0.50
VERL C-unique	1021	855	17	8	0.0036	0.0033	−0.26	0.56	−3.27	0.040	0.47
non-reproductive loci											
nuclear *rib*	498	0	10	n/a	0.0044	0.0028	−1.01	−0.55	0.85	0.059	n/a
nuclear *rtp*	459	0	12	n/a	0.0057	0.0030	−1.38	0.25	1.10	0.068	n/a
nuclear *eef*	132	0	1	n/a	0.0017	0.0008	−0.69	0.53	0.10	0.066	n/a
mito. *COI*	473	473	2	0	0.0011	0.0010	−0.22	−0.76	−0.71	0.089	0.04

N = 28 individuals. nt = nucleotides, cds = coding sequence, S = polymorphic sites, S_N_ = nonsynonymous polymorphic sites, θ_W_ = Watterson's theta, π = nucleotide diversity, D_Taj_ = Tajima's D, D_FL_ = Fu & Li's D, H_FW_ = Fay & Wu's H, an asterisk(*) denotes statistical significance at α = 0.05, K_JC_ = Jukes-Cantor corrected distance to outgroup, *d*_N_/*d*_S_ = nonsynonymous/synonymous divergence.

All five exons of lysin were sequenced in both species, except for a portion encoding part of the secretion signal. For pink abalone, most of the intron sequence was also obtained, yielding an alignment of almost 6 kilobase pairs (kb) of the lysin gene region. In green abalone, a subset of lysin introns was sequenced, yielding more than 2 kb aligned in total. Notably, neither abalone species contained non-synonymous polymorphisms in lysin; however, there were synonymous and intronic polymorphisms. Levels of polymorphism in lysin were lower than at non-reproductive, nuclear loci for both species (Pink: θ_W_(lysin) = 0.0014, θ_W_(non-rep) = 0.0046; Green: θ_W_(lysin) = 0.0007, θ_W_(non-rep) = 0.0081; Table 1 and Table 2). The non-reproductive theta estimates above were made from a concatenation of the nuclear, non-reproductive loci. The low levels of polymorphism at lysin suggest recent positive selective sweeps, which we will explore in a later section.

### Lysin and VERL Are Not Closely Linked

We first asked whether lysin and VERL were physically linked. We used a mapping family of *Haliotis discus*, a species closely related to both study populations. The wild-caught male parent was mated to a female from a cultivated strain as previously described [26]. The male parent contained polymorphisms in both lysin and VERL allowing us to score recombination in the progeny. Of 20 genotyped progeny, we observed 11 of the parental male genotype versus 9 recombinants. By examining the log odds ratio (LOD) for linkage given the progeny genotypes, we can confidently reject linkage between lysin and VERL at distances of 17 centiMorgans (cM) or less (LOD threshold of −2.0) (Section 11.3 of Strachan and Read [27]). Therefore, there is a great deal of recombination between them and we should not expect linkage disequilibrium simply due to physical linkage. In fact, the progeny genotypes are consistent with independent assortment between lysin and VERL.

### Association between Lysin and VERL Alleles

**Figure 2:**
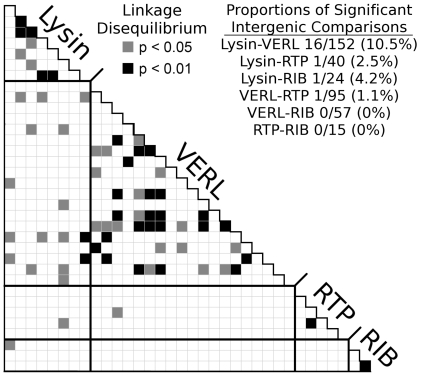
Linkage disequilibrium between tagSNPs in lysin and VERL. A triangle plot shows the significance of linkage disequilibrium (LD) within and between genes in the pink abalone. Non-reproductive genes (RTP and RIB) were included as negative controls. We considered only a set of tag SNPs with minor allele frequencies of 5% or greater. As expected, there is a large amount of intragenic LD and little between physically unlinked SNPs (intergenic). However, there is a significant excess of LD between lysin and VERL (P = 0.0041, binomial test).

We studied patterns of linkage disequilibrium (LD) between lysin and VERL to test for a genetic association between them, which could result from strong selection for compatible alleles. We studied the pink abalone population because it contained sufficient polymorphisms in lysin, VERL, and non-reproductive genes. The green abalone could not be studied because it contained only one polymorphism in VERL. The strength of LD between any pair of genes was judged by the proportion of single nucleotide polymorphism (SNP) comparisons showing statistically significant LD (α = 0.05). Because several polymorphisms within each gene were in LD due to physical proximity we chose a single “tag SNP” to represent each block of associated SNPs [28]. By analyzing tag SNPs, intragenic LD was reduced and single tests between intergenic SNPs became more independent. For example, the average r^2^ between all lysin SNPs is 0.228, and after choosing tag SNPs it was reduced to 0.105. LD between any pair of tag SNPs was assessed by a test for genotype data, and significance was determined by comparison to a distribution of random permutations [29]. To improve power, we excluded rare SNPs with minor allele frequencies less than 5% [30]. As expected, many intragenic comparisons showed significant LD due to physical linkage (Figure 2). In contrast, we do not normally expect LD between unlinked genes. Comparisons between non-interacting genes served as a control against genome-wide LD, which could result from cryptic population structure or migration. Only 3 of the 231 comparisons (1.3%) between non-interacting genes were significant; however, 16 of the 152 comparisons (10.5%) between lysin and VERL were significant (Figure 2). This proportion between lysin and VERL exceeds the nominal rate of the test (α = 5%). Because comparisons between tag SNPs are more independent, we can use the binomial distribution to show that this proportion was not likely to occur by chance (P = 0.0041, 16 or more successes in 152 trials). We performed these tests again comparing only those SNPs with similar allele frequencies because the comparison of frequency-matched SNPs can be a more sensitive measure of LD [30]. The results were similar: 9 of 110 lysin-VERL comparisons (8.2%) and 2 of 183 non-interacting comparisons (1.1%) were significant.

Although the tag SNPs reduced intragenic LD, the tests were still not completely independent, and remaining LD could lead to correlated results and departures from the nominal rate of the test. Generally, tag SNPs are chosen using a threshold level of r^2^ to group associated SNPs. As this threshold is dropped, groups will become larger, the number of tag SNPs will decrease, and intragenic LD will decrease. For example, using a threshold of 0.1, average r^2^ within lysin drops from 0.228 to 0.048. To study the robustness of the lysin-VERL association to varying levels of intragenic LD we repeated our tag SNP analysis at a series of lower thresholds starting from the initial level of 0.64, and decreasing to thresholds of 0.4, 0.3, and 0.1. As the threshold dropped, the proportion of significant comparisons between lysin and VERL always significantly exceeded the nominal rate, and actually increased as intragenic LD decreased (Table 3). In contrast, the proportion of significant non-interacting comparisons remained between 1 and 2% across all thresholds. Because we showed above that lysin and VERL are not closely physically linked, the observed LD between lysin and VERL is consistent with strong selection for compatible alleles.

**Table 3:** 

tag SNP threshold (r^2^)	Significant Intergenic Comparisons			Intragenic LD: mean r^2^			
	Non-interacting	Lysin-VERL	P-value	Lysin	VERL	*rtp*	*rib*
0.64	3 of 231 (1.3%)	16 of 152 (10.5%)	0.0041	0.105	0.094	0.078	0.077
0.4	2 of 145 (1.4%)	10 of 78 (12.8%)	0.0055	0.076	0.102	0.027	0.077
0.3	2 of 138 (1.4%)	9 of 72 (12.5%)	0.0096	0.076	0.095	0.027	0.077
0.1	1 of 76 (1.3%)	5 of 33 (15.1%)	0.0230	0.048	0.085	0.005	0.009

### Rapid Amino Acid Divergence in VERL

We first tested for positive selection using both divergence and polymorphism data with the McDonald-Kreitman test (MK), which compares the abundances of non-synonymous and synonymous substitutions to non-synonymous and synonymous polymorphisms [31]. The MK test indicated a significant excess of non-synonymous divergence in the first two repeats between pink abalone and an outgroup species, *H. discus*. (P = 0.012). This constitutes evidence for long-term positive selection for amino acid substitutions in the first two repeats and corroborates the inference of recurrent positive selection in these repeats previously made using only divergence data [21]. In contrast, the last repeat in pink abalone did not show a significant excess of non-synonymous divergence. In the green population, the MK test could not be performed for VERL because only 1 polymorphism was observed.

### Polymorphism-Based Tests of Selection on Pink VERL

**Figure 3:**
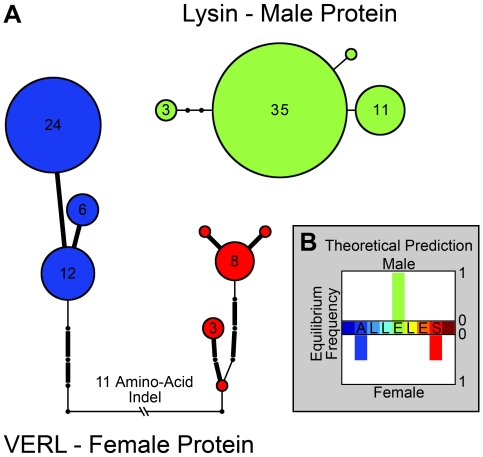
Polymorphisms in lysin and VERL match a unique prediction of sexual conflict. (A) In these haplotype networks, each pink abalone haplotype is a node with size proportional to allele frequency. Absolute frequencies are given inside each node. Edges between nodes are substitutions; edges representing nonsynonymous substitutions are in bold. The lysin network is of the largest region (∼1 kb) with no observed recombination events. Notably, there are no amino acid polymorphisms in the entire coding sequence of lysin. The VERL network was constructed using all 306 resequenced nucleotides in the last repeat. The two divergent clades of VERL alleles do not reject Hardy-Weinberg equilibrium. (B) Models of sexual conflict predict that female diversification can reduce rates of polyspermy. One possible outcome of these models is diagrammed. Bars represent the predicted equilibrium frequencies of alleles in males and females. Maximally compatible alleles have the same value (color) on the x-axis (ALLELES). Males are prevented from specializing on one group of females by frequency dependent selection. Shifting the mean male phenotype toward either group of females results in a benefit to and increase in the frequency of alleles in the other group of females. Diagram adapted from Gavrilets and Waxman [58].

While the divergence-based tests above reflect long-term patterns of evolution, polymorphism data can shed light on recent, population-specific evolutionary events. We first present results for VERL in the pink population. We analyzed the N- and C-terminal portions of VERL separately because they had very different levels of polymorphism and they are separated by more than 8 kb. The N-terminal portion consisted of the first 2 repeats in the repeat array, while the C-terminus consisted of the last repeat and some non-repetitive C-terminal sequence. We first studied the polymorphism frequency spectrum at these regions to test for departures from neutral evolution (Table 2). In the first 2 repeats, the statistics Tajima's D, Fu & Li's D, and Fay & Wu's H were not significantly different from the neutral expectation [32]–[34]. However, the last repeat of VERL in the pink abalone showed a significantly positive Fu and Li's D statistic, due to an excess of substitutions on internal branches (Table 2) (P = 0.021). This result is consistent with balancing selection favoring the maintenance of divergent lineages in the population. We can see the potential effects of such selection in the relationship of the observed alleles (Figure S1). For example, alleles of the last repeat are deeply subdivided into two clades separated by an average of 3.9 and a maximum of 10 amino acid substitutions and by an indel of 11 amino acids (Figure 3A). The amount of polymorphism between these alleles exceeds divergence between some pairs of abalone species (Figure 1), because there were only 3 fixed substitutions between pinks and *H. cracherodii* and 6 to *H. discus*. Generally, the last repeat contains more polymorphism than all of the non-reproductive loci (Table 2), although not significantly more (HKA test, P = 0.057). Tajima's D also deviates in the direction expected for balancing selection, although its deviation was not significant (D = 1.06, P = 0.111).

Population history, such as structure or growth, can affect patterns of polymorphism and hence influence these statistical tests of neutrality; however, such demographic events would have a general effect on all loci in the genome. We applied the frequency spectrum tests used above to the three nuclear, non-reproductive genes in pinks and confirmed that VERL's deviation from neutrality was locus-specific (Table 2).

### Probing VERL's Internal Repeat Array

An alternative explanation for the observed deep divergence is that concerted evolution brought a rare interior repeat to the last repeat, since interior repeat units could harbor divergent copies. However, the array is thought to be homogenized by concerted evolution, which involves unequal crossing over and/or gene conversion [35]. We looked deeper into the array to get a better picture of this process in pink VERL. We performed a Southern blot to examine 2 pink individuals for sequence divergence among the interior repeat units. First, we excised the VERL repeat array from genomic DNA with restriction enzymes that cut in the second repeat and downstream of the last repeat. We then performed a partial digest in the repeats to determine the repeat types found in the interior. Because we saw two divergent clades of last repeats, we chose a restriction enzyme, *Bpu*10I, which distinguishes between the two clades. Specifically, *Bpu*10I cuts in the 33 bp insertion that separates the clades. After performing a partial digest with *Bpu*10I we probed the non-repetitive C-terminal end of the resulting fragments. If the interior repeats were of the clade containing the insertion, the partial digest should produce a ladder at specific fragment sizes. If instead the array contains different internal repeats we should only see two bands, one at low molecular weight and another at high molecular weight (uncut by *Bpu*10I). We also searched for sequence divergence with a *Bsm*I partial digest, which should cut at a site in both clades.

We performed the partial digests on 2 individuals: one a homozygote at the last repeat for the insertion clade, the other was heterozygous for the two clades. Our sample contained no individuals homozygous for the deletion clade. Individual pink35 produced a ladder of 5 visible bands at the predicted molecular weights for *Bpu*10I lanes 2&3 (Figure S2). The 5 fragments were the exact sizes predicted for a single cut in each repeat unit upstream of the last repeat. While the ladder of repeats should continue up to about 20, we did not expect to see the full ladder because each fragment's abundance decreases with its distance from the probe. The 5 visible bands show that the insertion clade repeat is also found in the 4 immediately upstream repeats. Individual pink36 showed similar bands except the second band (Figure S2: “d”) was missing. This indicates either a different repeat clade or a nucleotide substitution in the restriction site. For both individuals the *Bsm*I lanes showed ladders as expected and did not show any evidence for interior repeat divergence. Notably, lane 4 resolved a very large repeat ladder with no evidence for divergence. Overall, the partial digests show that the insertion repeat clade is found in the interior of the array as well as at the last repeat, and that there is some evidence for sequence divergence in pink36. Also of note, these individuals showed different full repeat array sizes, which are apparent in the non-partially digested lanes (“1”). The band for pink35 is just above the 10 kb marker, which corresponds to a slightly truncated array of approximately 20 units, while individual pink36 is at 11.1 kb and approximately 22 units. Such polymorphism in array lengths is expected if unequal crossing-over is occurring.

### Population Tests of Positive Selection on Green VERL

VERL in the green population showed only 1 polymorphism in 1,470 aligned basepairs. HKA tests showed that such low polymorphism is not consistent with the expected neutral level of polymorphism. These HKA tests compared both N- and C- terminal VERL regions with each of 3 non-reproductive loci (*rib*, *cellulase*, and *COI*). All comparisons indicated a significant deficiency of polymorphism in VERL: VERL N-terminus *vs. rib*, *cellulase*, and *COI*: P = 0.018, P = 3.0×10^−5^, and P = 5.3×10^−8^, respectively; VERL C-terminus: P = 0.039, P = 0.0016, and P = 4.1×10^−5^, respectively. The 2 HKA tests above which compared VERL to mitochondrial *COI* were appropriately corrected for the haploid, maternal inheritance of the mitochondrion. This strong reduction suggests a recent selective sweep affecting both termini of green VERL, but unfortunately the lack of polymorphisms also precludes the application of further tests.

### Tests of Positive Selection on Lysin

Using both divergence and polymorphism data, we observed an excess of non-synonymous divergence in lysin for both pink and green populations (MK tests: P = 0.006 and P = 0.00044, respectively). This result indicates recurrent positive selection for amino acid changes over long time periods, and is consistent with past divergence studies.

**Figure 4:**
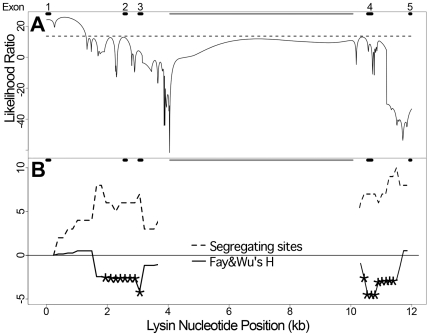
A recent selective sweep at exon 1 of lysin. (A) A profile of the likelihood (y-axis) that a selective sweep originated at each site across the lysin gene region (x-axis). Values near exon 1 exceed the significance threshold (α = 0.01, dashed line). Black bars at the top of the plot denote exons. The thin, grey bar spans an unsequenced portion (∼6 kb) of intron 3. (B) Sliding window of polymorphism (Segregating sites) and Fay&Wu's H statistic plotted along the lysin gene region (window size = 1,000 bp, step size = 100 bp). Polymorphism near exon 1 is close to zero and recovers as the sliding window moves in the 3′ direction, consistent with a recent sweep originating in exon 1. The H statistic indicates an excess of high frequency derived alleles downstream of the putative sweep target, as predicted for genetic hitchhiking with a selective sweep. An asterisk denotes a significant rejection of the neutral model for the H value in that window (α = 0.05).

We tested the hypothesis of a recent selective sweep at lysin using polymorphism-based methods. Lysin in both populations is characterized by the presence of a single coding allele and by abnormally low levels of silent polymorphism. HKA tests compared polymorphism and divergence between lysin and non-reproductive loci in both species and suggested that these reductions in polymorphism are non-neutral. In pink abalone, 2 nuclear loci indicated a significant reduction at lysin (lysin *vs. rib*, P = 0.025, lysin *vs. rtp*, P = 0.0074), while 1 small nuclear locus, *eef*, did not. The mitochondrial locus *COI* has roughly the same level of polymorphism as lysin; however, it is more reasonable to compare lysin (θ_W_ = 0.0014) with *COI* synonymous site polymorphism (θ_Wsyn_ = 0.0042) because most of lysin's sequenced region is non-coding. In green abalone, polymorphism at lysin is significantly lower than all non-reproductive loci examined (lysin *vs. rib*, P = 0.0065; lysin *vs. cellulase*, P = 2.2×10^−9^; lysin *vs. COI*, P = 5.8×10^−12^). The departure from neutrality between lysin and the non-reproductive loci could be due to either a deficiency of polymorphism at lysin or an excess of divergence in lysin's introns. Estimates of divergence are similar for introns of lysin and the non-reproductive loci (Table 1 and Table 2, K_JC_), and there is no reason to expect divergent selection in lysin's introns. Therefore, polymorphism was likely reduced in the lysin gene region by a non-neutral process, such as a recent selective sweep. Overall, green lysin showed a strong reduction in polymorphism and is consistent with a sweep across the entire region. Pink lysin showed a more moderate reduction across the whole gene, but sliding window analysis shows a strong local reduction around exon 1 (Figure 4B).

Summary statistics of the polymorphism frequency spectrum (Tajima's D, Fu & Li's D, and Fay & Wu's H) were not significantly different from the neutral expectation in either species when calculated over the entire gene region (Table 1 and Table 2); however, in pink lysin a sliding window analysis of Fay & Wu's H statistic rejects neutrality from 2 kb downstream of exon 1 until the end of the sequenced region (Figure 4B). Following a sweep, genetic hitchhiking is predicted to form an excess of high frequency derived polymorphisms (indicated by a low value of H) flanking a region of strongly reduced polymorphism [34],[36]. Interestingly, this is what we observed at pink lysin. Frequency-based tests did not reveal evidence for selection at green lysin; however, this does not exclude its possibility because these statistics typically demonstrate low power and detect departures only within specific time periods after a sweep [37]–[39].

A composite likelihood test that takes advantage of our nearly 6 kb of contiguous sequence in pink abalone revealed further evidence of a recent selective sweep. (This test was not performed in greens because that population only contained 6 SNPs in lysin.) The composite likelihood method evaluates local patterns of polymorphism for consistency with a selective sweep model by considering the locations and allele frequencies of the observed polymorphisms [40]. We followed the published composite likelihood procedure by Kim and Stephan for testing a particular region using their software. The likelihood ratio statistic, LR1, which reflects the fit of the data to a selective sweep model, was first computed for the pink lysin data. The statistical significance of this LR1 value was determined by comparison to those of 1,000 data sets simulated without a selective sweep but under population parameters inferred from the observed data. We used experimental data to estimate the recombination rate (4N_e_r = 6.02×10^−3^; see Methods) and baseline polymorphism level (θ_W_ = 0.0046; pooled nuclear, non-reproductive loci). The inference of a selective sweep at pink lysin was highly significant (P<0.001) as no simulated dataset had an LR1 value more extreme than that of the observed data. To test the robustness of this inference to population demographics, we performed a goodness-of-fit test (GOF) to a selective sweep model using the method of Jensen *et al.* [41]. This is an important control because population demographics, such as structure, can create signatures that resemble a selective sweep. A goodness-of-fit statistic (GOF1) evaluated the fit of our lysin data to a selective sweep model when compared with an alternative, parameter-rich model. We then compared the observed GOF1 value to 1,000 data sets simulated under a selective sweep with parameters matching those inferred from the data. The GOF test does not reject a selective sweep model for an alternative, parameter-rich model (P = 0.790), which suggests that the selective sweep signature is not due to population demographics. Finally, we used a sliding window profile of the LR1 statistic to infer the genic regions containing the selected mutation. This is inferred where the statistic crosses a significance threshold of (P = 0.01). The profile indicated that the sweep was likely due to a selected mutation at or near exon 1 (Figure 4A). Indeed, nucleotide polymorphism is near zero at exon 1 and steadily increases downstream, consistent with polymorphisms recombining onto the selected haplotype during a sweep (Figure 4B) [36]. We inferred the amino acid substitutions leading from a recent ancestor to the extant pink lysin sequence, and 5 of the 10 total substitutions were within exon 1, any of which could have been responsible for the inferred selective sweep. Notably, previous divergence studies also indicated several codons in exon 1 as frequent targets of positive selection [22],[42].

### Coevolution along Phylogenetic Lineages

**Figure 5:**
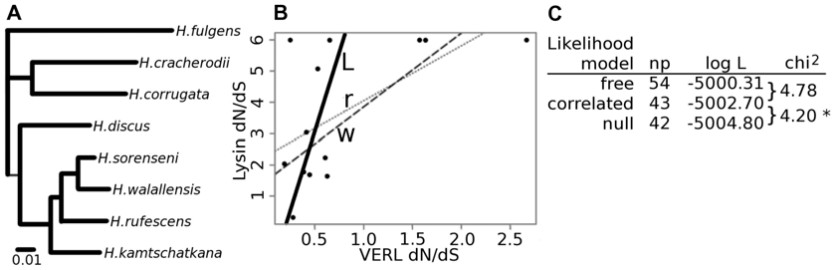
Evolutionary rates at lysin and VERL correlate over an extended timescale. (A) Lysin and VERL *d*_N_/*d*_S_ values were estimated on each branch of an 8 species phylogeny. (B) Lysin *d*_N_/*d*_S_ point estimates are plotted versus VERL for all 13 branches. Note the difference in scale between the axes. Regression lines are plotted for each of 3 methods: a standard linear regression (‘r’, dotted line), a weighted linear regression (‘w’, dashed line), and the line described by our ‘correlated’ likelihood model (‘L’, solid line). (C) Results of our likelihood models. Each model is shown with its number of free parameters (np) and maximum log likelihood (log L). Comparisons between models were made using a likelihood ratio test (LRT). An asterisk (*) indicates a statistically significant difference between models at α = 0.05.

We evaluated coevolution between lysin and VERL over long timescales by testing for a correlation of evolutionary rates during the divergence between species. Our expectation was that rates of amino acid evolution for lysin and VERL would co-vary due to compensatory evolution and shared selective pressures. Hence, *d*_N_/*d*_S_, a measure of evolutionary rate, would correlate between corresponding branches of VERL and lysin phylogenies. We took advantage of 8 closely related species from the north Pacific, including the pink and green species, for which confident sequence alignments could be made and which represent levels of divergence appropriate for the use of codon models (*Haliotis corrugata*, *H. cracherodii*, *H. discus*, *H. fulgens*, *H. kamtschatkana*, *H. rufescens*, *H. sorenseni*, and *H. walallensis*). Importantly, synonymous sites are not saturated between these species; for example, the estimated divergence at synonymous sites (*d*_S_) over the longest branch is 0.11. We determined a phylogenic tree for these species using the coding sequence of lysin and the first 3 repeats of VERL. Using a Bayesian method, one tree topology had a posterior probability greater than 99% and was used to represent the species phylogeny over which the proteins would coevolve (Figure 5A).

We tested for a correlation of *d*_N_/*d*_S_ values using several methods. As a first, simple approach we made *d*_N_/*d*_S_ estimates for each branch using the branch model of *codeml* [22] and analyzed them with linear regression models. Because some short branches had no synonymous changes in lysin, their *d*_N_/*d*_S_ ratios are undefined but *codeml* assigns an arbitrary value of 999. These branches were a challenge to using conventional regression models, and to lessen their effect on the regression, we set their *d*_N_/*d*_S_ values equal to 6, the nearest integer above the largest estimated value. The linear regression of lysin on VERL *d*_N_/*d*_S_ point estimates yielded the predicted positive relationship (r^2^ = 0.35) (Figure 5B: dotted line ‘r’) and was statistically significant (F_1,11_ = 5.83, P = 0.034). Because the extreme *d*_N_/*d*_S_ values on short branches could have a strong influence on this correlation, we also performed the regression after removing those branches with fewer than 5 total substitutions in lysin. The correlation became stronger (r^2^ = 0.51) and remained statistically significant (F_1,8_ = 8.477, P = 0.020). Also, to better account for the uncertainty in each *d*_N_/*d*_S_ estimate we performed a weighted linear regression with the relative amount of divergence on each branch as a weighting factor. The weighted regression was also positive and significant (r^2^ = 0.40, F_1,11_ = 7.34, P = 0.020) (Figure 5B: dashed line ‘w’). Although these initial results were encouraging, we found them unsatisfactory because the strength of the correlation depended on which corrections were implemented and which value was chosen to limit the extreme *d*_N_/*d*_S_ estimates. Ideally, we wanted a method that would evaluate the correlation while using a model of sequence evolution to account for uncertainty in *d*_N_/*d*_S_. To this end, we developed a novel method to test the correlation entirely within the phylogenetic model, instead of using point estimates.

We created likelihood models in which the evolutionary parameters for both proteins were jointly estimated using the program HyPhy [43]. Our most general model, ‘free’, allows each branch of both genes trees to have its own *d*_N_/*d*_S_ value. The ‘free’ model is analogous to two branch models of *codeml*, one for each gene. Next, the ‘correlated’ model constrains *d*_N_/*d*_S_ values to fall on a line of correlation for corresponding branches of lysin and VERL. This line is described by 2 global parameters representing its slope and y-intercept. When optimized, the ‘correlated’ model's likelihood reflects the strength of correlation. The ‘null’ model also models *d*_N_/*d*_S_ values on a line, but its line is constrained to have a slope of zero, representing an uncorrelated relationship between lysin and VERL. A likelihood ratio test between the ‘correlated’ and ‘null’ models tests whether the slope of the correlation line is significantly different from zero.

The optimized results for these models are shown in Figure 5. The ‘correlated’ model described a line closely fitting the *d*_N_/*d*_S_ values of the longer, more informative branches, because the weighting of these branches was inherently higher in the evolutionary model (Figure 5B: solid line ‘L’). Similarly, the effect of the extreme, undefined values was much less. The ‘correlated’ model fits the data significantly better than the ‘null’ model (LRT = 4.2, P = 0.040; Figure 5C). Also, we found that the ‘correlated’ model described the data well because the ‘free’ model was not significantly better (P = 0.94; Figure 5C). Since the assumed species tree could influence the outcome of these tests, we explored alternative topologies, including the lysin gene tree, a tree containing polytomies where lysin and VERL trees disagree, and the consensus tree using a maximum likelihood method. (The VERL tree topology did not differ from the Bayesian-determined topology used initially.) All of these topologies were largely in agreement, and the few differences were between 4 closely related species (the bottom 4 leaves in Figure 5A). For each of these alternative topologies the ‘correlated’ model rejected the ‘null’ model (P = 0.021, 0.021, 0.048, respectively). In summary, multiple approaches indicated a positive and significant dependency between lysin and VERL *d*_N_/*d*_S_ values during the divergence of these 8 species, reflecting long-term coevolution.

To test the robustness of these results, we performed the likelihood test on simulated sequences and between non-interacting abalone proteins. We first simulated “pseudo-VERL” sequences by shuffling its *d*_N_/*d*_S_ values among branches and then simulating along the species tree. We could not perform the same shuffled simulation for lysin because of its undefined *d*_N_/*d*_S_ estimates. We also simulated VERL and lysin sequences by pulling random branch *d*_N_/*d*_S_ values from a gamma distribution while maintaining all other parameters from the observed data. These 3 sets of simulated sequences were then tested for correlated evolution as described above. The shuffled VERL datasets only showed coevolution with lysin that met or exceeded the real data in 1 of 1,000 simulations. Similarly, the gamma-distributed VERL datasets exceeded the observed statistic in only 2 of 1,000 simulated sets. The simulated lysin sequences showed coevolution with VERL that matched or exceeded the observed data in 148 of 1,000 sets. These bootstrap analyses suggest the following p-values for the observed correlation: 0.002, 0.003, and 0.148, respectively. We then sequenced two control genes, *cellulase* and *haemocyanin*, in all eight species and tested their correlation with lysin and VERL and with each other. We did not expect correlated rates for these five non-interacting comparisons. None of them were statistically significant, and their LRT statistics were 1.52, 2.78, 1.34, 2.08, and 0.40, compared with 4.20 between lysin and VERL.

## Discussion

### Signatures of Coevolution between Lysin and VERL

We used the well-characterized interaction between lysin and VERL to investigate two potential signatures of coevolution. The first signature is the prediction that compatible alleles of lysin and VERL would be associated within individuals. We saw significant signs of association between lysin and VERL but not between non-interacting genes. Such tests between genes require measures to reduce intragenic linkage disequilibrium (LD) so that individual SNP comparisons will be more independent. This is a significant challenge as intragenic LD can never be completely removed. However, it can be reduced to low levels by using a tag SNP to represent each block of associated SNPs. We chose multiple sets of tag SNPs to yield lower and lower levels of residual intragenic LD. We found that the proportion of significant comparisons between lysin and VERL remained high and statistically significant for all of these sets (Table 3). This observation supports an association between a sexually selected trait and preference for that trait, in which the gene controlling preference (VERL) would select compatible alleles of the male trait (lysin). This type of selection is a required step in the coevolution of the two genes. An alternative explanation for the association is that lysin and VERL are physically linked; however, to create the observed association via physical linkage, theory predicts that they would need to be very closely linked, at a genetic distance of approximately 0.005 cM. Given the results of our progeny array, the LOD score for this distance is less than −33, and linkage at distances up to 17 cM are ruled out using a LOD threshold of −2. Hence, physical linkage is not at all likely to be the cause of the observed association between lysin and VERL alleles. Finally, it seems that the observed association between lysin and VERL alleles formed prior to the latest selective sweep in lysin since it currently contains no amino acid polymorphisms. According to this hypothesis we observed the residual LD between lysin SNPs that recombined onto the swept polymorphism. Indeed, the SNPs in LD with VERL are in the downstream portion of the lysin gene, away from the location of the putative selective sweep.

The second potential signature of coevolution is a correlation in the rate of amino acid substitution (*d*_N_/*d*_S_) along phylogenetic branches. A correlation is predicted because as one protein experiences an increase in its evolutionary rate, perhaps due to an adaptive episode, the rate of compensatory evolution in its coevolving partner would also increase. In addition, evolutionary pressures acting on both proteins could create a correlation, such as a change in the strength of functional constraint. We considered multiple approaches to test correlated evolution between lysin and VERL: a linear regression of *d*_N_/*d*_S_ point estimates, a weighted regression of point estimates, and a novel set of likelihood models. Each of these approaches showed a positive and significant correlation between lysin and VERL *d*_N_/*d*_S_ values; however, we consider the results of the likelihood method to be the most compelling for several reasons. The likelihood method uses an evolutionary model, so it weighs each branch proportionally to the certainty in its *d*_N_/*d*_S_ value. Second, the branches of lysin without synonymous substitutions and the arbitrarily chosen limit to *d*_N_/*d*_S_ had strong effects on the linear regressions. These various approaches all showed a positive and significant correlation between lysin and VERL evolutionary rates. We also performed several control comparisons to test whether the observed level of correlation between lysin and VERL was unique. Our VERL simulations showed that a correlation of the strength observed between lysin and VERL was rarely observed using random *d*_N_/*d*_S_ values or shuffling of observed values; however, the simulated lysin sequences showed correlations with VERL in about 15% of cases. The cause for this discrepancy between simulated loci is not clear, but it could be an effect of lysin's small size. The lysin simulations cause us to recommend some caution to the conclusion of phylogenetic coevolution. The five comparisons between non-interacting abalone proteins did not show any significant correlations and were not comparable to that between lysin and VERL, so there does not seem to be a genome-wide correlation between proteins in these species. Such a genome-wide correlation could be expected if there were large changes in effective population size along branches. These results support the uniqueness of lysin-VERL coevolution. In general, we found a significant and positive relationship between lysin and VERL evolutionary rates, as predicted under coevolution. In the future it will be interesting to test for such a correlation within other species and between other interacting fertilization proteins.

Although general studies of protein networks have revealed correlated rates of evolution for interacting proteins [9],[10],[44], there is debate over how much of the correlation can be attributed to coevolution at the interaction interface or, instead, to shared selective pressures [45]. In the case of lysin and VERL, their divergence is driven predominantly at their sites of interaction, which are the N- and C- termini of lysin and the repeat units of VERL. Therefore, the correlation between lysin and VERL is more likely due to coevolution between their interacting regions. The ‘correlated’ likelihood model can tell us about the relative rates of evolution between the two proteins. Coevolution could proceed through relatively equal numbers of changes to each protein. Alternatively, multiple compensatory substitutions could occur in one protein for each single change in the other. The ‘correlated’ model suggests that the latter relationship exists between lysin and VERL. The slope of the correlation line was approximately 10, reflecting a greater number of amino acid changes in lysin for each change to VERL. However, this value is also expected to be influenced by the strength of negative selection (conservation) on each protein, and so should not be interpreted as the sole effect of compensatory changes. This difference of evolutionary rates suggests that in order to maintain optimal binding, several changes in lysin are required for each change in VERL, a possibility also discussed by Nei and Zhang [46].

### Polymorphisms at Lysin and VERL Indicate Recent Selection

Previous studies used divergence between species to infer positive selection on lysin and VERL and to specify the individual codon sites where it acted [21],[22],[47]. Our polymorphism data independently support the inference of positive selection on both proteins and even indicate its action at the same structural regions. Polymorphism at lysin was reduced in both pink and green populations, consistent with recent selective sweeps. Selective sweeps at lysin could be very frequent, because in all four species in which a population has been studied, levels of polymorphism are seriously reduced and not a single amino acid polymorphism has been found: *Haliotis corrugata* and *H. fulgens* (this study), *H. tuberculata* [48], and *H. rufescens* [47]. The lack of amino acid polymorphism is remarkable considering lysin's extreme rate of divergence [47]. In this study, the composite likelihood method inferred a selective sweep encompassing the first exon of pink lysin, in agreement with the codons frequently inferred under selection using divergence data [22] and consistent with experimentally determined species-specific domains discovered through site-directed mutagenesis [18]. This agreement is a nice example of functional data correlating with evolutionary analyses. Similar to lysin, VERL in green abalone may have undergone a recent selective sweep because there was only 1 polymorphism among 32 chromosomes, and such a severe reduction in polymorphism over 1,470 bp was inconsistent with neutral evolution. VERL in the pink abalone was more complex. When we looked at divergence and polymorphism together using the McDonald-Kreitman test, we saw a significant excess of non-synonymous divergence at repeats 1 and 2. This result corroborates *d*_N_/*d*_S_ analyses in which recurrent positive selection was inferred within the first 2 repeats but not in the remainder of VERL [21]. Polymorphism-based evidence alone sheds light on recent, population-specific evolution at pink VERL. While the first 2 repeats showed no polymorphism-based evidence of recent selection, the last repeat of pink VERL showed a signature different from all other analyzed regions. It had a high level of polymorphism, and alleles were divided into 2 clades, each separated by multiple amino acid changes and an 11-amino acid indel (Figure 3A). Such high diversity could be created as a result of frequency-dependent or balancing selection, as is suggested by the frequency spectrum tests. An alternative explanation could be that the array contains divergent internal repeats, one of which moved to the last repeat by gene conversion. We also consider this a plausible alternative; however, three experimental results lead us to prefer selection. The question is whether the rate of array homogenization via concerted evolution is great enough to suppress deep divergence among internal repeats. One allele of VERL in *H. rufescens* has been completely sequenced. All of its repeats 3 through 22 showed at least 95% nucleotide identity, and hence did not contain divergent interior repeats [23]. Another study randomly cloned interior repeats from several species, and the repeat units were closely related [35]. Finally, our Southern blot did not reveal great divergence in the interior repeats. It will take additional in-depth studies of VERL alleles to better distinguish between these two hypotheses. While previous divergence studies did not infer positive selection in repeats 3 through 22 [21], our observations in pink and green abalone suggest more complex evolution, possibly involving selective episodes.

### Sexual Conflict and the Evolution of Fertilization Proteins

Several hypotheses are offered to explain the rapid divergence of fertilization proteins [2]. One such hypothesis, sexual conflict, describes a scenario in which the adaptive optimum of a trait differs between males and females [49]. When the conflict is mediated by different genes in males and females (interlocus sexual conflict), a continuous, adaptive struggle between male and female characters results. Indeed, experimental evolution in Drosophila has demonstrated that such antagonistic evolution between the sexes can occur as predicted [50]. For fertilization, there is a predicted sexual conflict over the fertilization rate and its effect on polyspermy. Polyspermy is the fertilization of an egg by multiple sperm, which in many species halts development. The optimum fertilization rate for a male is fast in order to outcompete sperm from other males [51], while the optimum for females is a moderate rate to control sperm entry and minimize the number of her eggs killed by polyspermy. Polyspermy increases when the rate of fertilization is too high because eggs cannot activate their defensive blocks before additional sperm fuse. There is evidence that the fertilization rate is not optimal for female abalone because their eggs suffer from polyspermy under natural sperm concentrations [52]. In addition, abalone have been observed in nature to spawn in aggregated groups and even in stacks of multiple individuals which would produce high local concentrations of sperm [53],[54]. Generally, polyspermy rates can be high in natural populations of free-spawning organisms, even under sperm-limited conditions [55],[56], and so the potential for conflict is great.

Polyspermy is directly related to fitness because it strongly affects the number of viable offspring, and hence a sexual conflict over fertilization rate would be strong. In theory, as the rate increases over generations due to sperm competition, the egg would counter-adapt to slow and regulate sperm entry. There is evidence that amino acid changes in lysin and VERL affect the fertilization rate in such a way. Lysin dissolves egg vitelline envelopes in a species-specific manner [15], and introducing lysin segments from one species into another has the corresponding effect on specificity [18]. While this experimental evidence shows the potential for lysin and VERL to control fertilization rate, and hence to mediate sexual conflict, theoretical predictions also corroborate a sexual conflict between lysin and VERL.

Mathematical models have predicted genetic outcomes within populations experiencing a sexual conflict [57]. The most common observation is a coevolutionary chase between female and male genes, involving recurrent adaptive evolution in both. An alternative outcome is also observed in which the female gene temporarily wins the conflict by diversifying and hence trapping the male gene at an intermediate position [58],[59]. In this situation, the female alleles form 2 distinct clades, and the male gene possesses a single allele that is equally adapted to each clade, yet suboptimal for either individually (Figure 3B). We found close resemblance between this stalemate outcome and alleles in the pink abalone; VERL last repeat alleles formed divergent clades separated by several protein differences (Figure 3A), and lysin showed only one coding allele. This stalemate outcome could also explain our hypothesis of balancing selection at VERL, because frequency-dependent selection would effectively maintain the two divergent clades. Notably, if we had studied only the male protein this pattern would have been missed. Generally, such a conflict over fertilization rate could be operating in diverse taxonomic groups because positive selection is widely observed at fertilization genes [60]. If conflict is widespread, it has great potential to drive the divergence of fertilization proteins and lead to reproductive isolation and speciation between allopatric populations. In theory, a sexual conflict could even drive speciation in a freely-mating (sympatric) population, because models show that the male gene could counter-diversify in response to the stalemate outcome, resulting in assortative mating [58].

## Materials and Methods

### Resequencing of Reproductive and Neutral Loci

*Haliotis corrugata* (pink) and *H. fulgens* (green) abalone DNA samples were isolated from non-destructive tentacle clips of individuals off Point Loma, San Diego, California, USA. Total DNA was isolated using the PUREGENE DNA purification kit (Gentra Systems, Inc, Minneapolis, Minnesota, United States). Genotypes were determined for VERL, lysin, and non-reproductive loci by PCR amplification from total genomic DNA followed by direct sequencing. We also sequenced these loci from *Haliotis discus* for use as an outgroup for *H. corrugata*, except for the first 2 repeats of VERL which were available in GenBank entry AF490763. PCR primers were designed from published transcripts of the lysin and VERL genes (Genbank M34389, M98875, AF490764, AF490763). We determined most of the lysin gene in pink abalone, including introns 1, 2, and 4. We also sequenced part of intron 3. The length of the unsequenced portion of intron 3 was estimated from a long-range PCR product spanning the region. Resequencing yielded almost 6 kb in each of 25 individuals for the pink lysin gene. In green abalone individuals, all exon and some flanking intron sequence of lysin was determined. Due to VERL's large repeat units (∼453 bp), only exterior repeats are accessible by PCR. Repeats 1, 2, and the C-terminal repeat were amplified by placing a primer outside the repeat array. Sequence coverage in pink and green abalone VERL was identical. For each pink individual, the number of 11-amino acid repeats between the larger repeats 1 and 2 was determined from the lengths of PCR products spanning this mini-repeat array. Introns of non-reproductive genes were amplified in pink abalone using PCR primers based on transcribed sequences from a *H. corrugata* ovary cDNA library [61]. Transcripts encoding highly conserved proteins were chosen to avoid loci potentially under positive selection. The intron-exon structure of these conserved genes was assumed identical to vertebrate homologs, and primers were placed within the boundaries of conserved exons. This resulted in successful PCR amplification of over half of attempted introns. The nuclear introns chosen for genotyping were from an abalone homolog of the oyster Ribosomal Protein L5 (*rib*), a homolog of Elongation Factor-2 (*eef*), and a homolog of Receptor Protein Tyrosine Phosphatase Delta (*rtp*). A mitochondrial locus, *cytochrome oxidase I* (*COI*), was amplified using published primers [47]. Four *H. fulgens* (green) *COI* sequences were retrieved from GenBank (AY679078-81). Our PCR primers are listed in the Supplementary Text S1, and PCR conditions are available from the authors upon request. Single band PCR products were sequenced on an ABI 3100 using Big Dye v.3.1 (Applied Biosystems, Foster City, California, United States). Sequence reads were called, aligned, and analyzed using phred, phrap, and consed [62],[63]. Polymorphisms were flagged by polyphred and visually confirmed [64]. Genotypes at each polymorphic site were verified manually, checked for Hardy-Weinberg equilibrium, and then transferred by Perl scripts to a phase-determining program, PHASE [65]. Control coding sequences for the phylogenetic coevolution method were sequenced from 8 species. *Cellulase* primers were designed from GenBank entry AB125892 which was also the source for the *H. discus* sequence. *Hemocyanin* coding sequences incorporated some of that produced by Streit *et al.* [66], and we performed additional sequencing using primers designed from GenBank entries AJ749644, AJ749646, AJ749647, AJ749648, AJ884595, AJ884596, and AJ252741. Gene regions newly sequenced in this study are available in GenBank entries FJ940228-FJ940677. The sequences for all 32 chromosomes at the C-terminal region of VERL in *H. fulgens* (greens) were identical to GenBank entry DQ453752 from nucleotide 85 to 774, and so they were not submitted.

### Linkage Disequilibrium

We chose a set of representative tag SNPs for each gene using the program ldSelect under the default parameters [28]. Gametic phase is unknown when testing for linkage disequilibrium (LD) between unlinked genes. To test LD in this situation we used a likelihood ratio test for genotype data implemented in Arlequin [67]. LD is tested for each pair of SNPs using a likelihood-ratio test which is compared to an empirical distribution generated by a permutation procedure [29]. This test assumes Hardy-Weinberg equilibrium, so sites that violated this assumption at the α = 0.01 level were removed from analysis. This LD test on genotypes performs well when the number of alleles is low; in our dataset, all SNP loci had only 2 alleles. We excluded rare variants below a minor allele frequency of 5%. In one test, frequency-matched SNPs were compared to improve power [30]. SNPs were considered frequency-matched if their minor allele frequencies were not more than 10% different, as recommended by Eberle *et al.* [30].

### Polymorphism and Polymorphic Tests of Selection

Summary statistics and sliding windows of polymorphism were calculated in DnaSP [68]. P-values for tests of selection were generated in DnaSP using coalescent simulations under the conservative assumption of no recombination. Hudson-Kreitman-Aguade (HKA) tests and McDonald-Kreitman tests were also performed in DnaSP. HKA tests comparing nuclear to mitochondrial loci in the green abalone used the appropriate correction for the haploid, maternal inheritance of the mitochondrion. Phylogenies of VERL segments in Figure 1 were determined and plotted using *dnaml* and *drawgram* of the PHYLIP package [69]. Haplotype networks in Figure 3 were created by TCS [70]. Lysin's haplotype network was based on the largest region without evidence of recombination between SNP markers (∼1 kb), and the network for the last repeat of VERL included our entire 306-basepair sequence of that region.

### Partial Digests and Southern Blot

We isolated genomic DNA from 2 individuals, one a homozygote for the last repeat type and the other a heterozygote. We first used two restriction enzymes to excise the VERL repeat array. These cut in the second repeat (*Bcl*I) and 1.6 kb downstream of the last repeat (*Bsa*I), and were allowed to cut to completion for 3 hours at 50°C. Partial digests were carried out for 2 different durations. We performed partial digests with *Bpu*10I at 37°C for 30 seconds and for 2 minutes. Partial digests with *Bsm*I were performed for 15 and 45 seconds at 65°C. All enzymes were obtained from New England Biolabs (Ipswich, Massachusetts, USA). Reactions were performed in 30 µl volumes with 2 µg genomic DNA each. We loaded 10 units (U) of each restriction enzyme except for *Bsm*I for which we used 3 U. Seven such reactions were run for each condition so that we digested 14 µg of genomic DNA for each lane. Reactions for each condition were pooled and the DNA was ethanol precipitated, which recovered about 8 µg for each lane. All recovered DNA was loaded into a 0.8% agarose gel and run for 15 hours at 40V in re-circulated TAE. DNA was blotted onto a nylon membrane using capillary action with ammonium acetate buffer as described in chapter 5.2 of Molecular Genetic Analysis of Populations [71]. Twenty-five ng of a 500 basepair PCR product of the C-terminal unique region of VERL was labelled with ^32^P by nick translation with a NEBlot kit (New England Biolabs, Ipswich, Massachusetts, USA). Hybridization was carried out at 65°C overnight followed by washes in standard saline citrate.

### Composite Likelihood Test

We performed a composite likelihood test for selective sweeps according to the procedure outline by the developers [40]. Selective sweep parameters and a composite likelihood statistic were inferred from the pink lysin data, and 1,000 neutral simulations were performed under parameter values estimated from the data. The values of the composite likelihood statistic from these simulations formed a null distribution against which to test a selective sweep in the data. Polymorphisms were polarized to ancestral and derived alleles using an outgroup sequence from *H. discus*. The average recombination rate in pink abalone was estimated from the sex-average genetic map length for a close relative, *H. discus* (2,320.1 cM) [72] and the measured genome size of pink abalone, *H. corrugata* (C-value = 2.00 pg: ∼1,956 Mb) [73]. These values yield a sex-average estimate of 1.19 cM/Mb across the pink abalone genome. Using the same method, our estimate for the human genome (1.02 cM/Mb) is near the generally agreed upon value (1.30 cM/Mb) [74]. We estimated the effective population size (N_e_) of pink abalone using the formula: N_e_ = θ/4μ. Polymorphism was estimated from the 3 nuclear, neutral loci (θ_W_ = 0.0046), and the nucleotide mutation rate per generation was taken from an experimental measurement in *C. elegans* (μ = 9.1×10^−9^) [75]. Hence our estimate of the recombination rate (4N_e_r) was 6.02×10^−3^ in pink abalone. The goodness of fit procedure for testing the robustness of an inferred selective sweep to population demographics was performed as prescribed [41]. The goodness of fit statistic (GOF1) for the sweep in pink lysin was compared to 1,000 simulations under parameter estimates for the inferred sweep.

### Coevolution by Correlated Selective Pressure

We tested the relationship between VERL and lysin *d*_N_/*d*_S_ ratios using linear regressions on point estimates and using novel likelihood models. Both approaches used multiple alignments of VERL and lysin from 8 species. The VERL alignment consisted of repeats 1, 2, and 3 as presented in Galindo *et al.* [21]. Lysin coding sequences for the same 8 species were retrieved from GenBank. A tree topology for these 8 species was determined by MrBayes using a concatenation of lysin and VERL and using the general reversible nucleotide model with rate variation modeled by the gamma function with a proportion of invariable sites [76],[77]. Linear regressions were performed using the ‘R’ program (http://www.r-project.org/) on branch *d*_N_/*d*_S_ values estimated by the *codeml* program of the PAML package [78]. Three joint likelihood models were created to evaluate the strength of correlation of branch-specific *d*_N_/*d*_S_ values of lysin and VERL. We constructed and maximized these models using a custom script written for HyPhy version 0.9920060106beta for Macintosh OSX [43]. The ‘free’ model is parameter-rich and has a separate *d*_N_/*d*_S_ value for each branch of each gene. The ‘free’ model will fit the data relatively well compared to the subsequent models. The ‘free’ model was created by partitioning the data into lysin and VERL and assigning a codon model and the tree topology determined above. Values of *d*_N_ and *d*_S_ were estimated using the Goldman and Yang codon model (GY94) [79]. The ‘correlated’ model was created to judge how well a correlated relationship explains the variation in branch *d*_N_/*d*_S_ values. It was created by placing the following constraint on each phylogenetic branch, *i*:
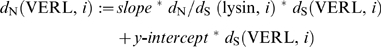

*Slope* and *y-intercept* are global parameters defining the correlation line (Figure 5B: solid line ‘L’). We optimized the ‘correlated’ model starting from 6 different initial values of the *slope* parameter to ensure convergence. The ‘null’ model was created to test if the correlation in the ‘correlated’ model is statistically significant. The ‘null’ model is defined by constraining the *slope* parameter to zero. Both VERL and lysin were analyzed as the dependent variable in the ‘null’ model and the maximum likelihood of the two was used. This step was necessary because both cases are uncorrelated and yet their maximum likelihoods are different. Finally, comparing the nested ‘correlated’ and ‘null’ models tests whether the slope parameter is significantly non-zero. A P-value for the rejection of the ‘null’ model in favor of the alternative ‘correlated’ model was obtained using a likelihood ratio test in which the twofold difference in their maximum log likelihoods is assumed to follow a χ^2^-distribution with degrees of freedom equal to the difference in the number of parameters. There is one degree of freedom between the ‘correlated’ and ‘null’ models. Model optimizations began with tree distances as initial parameter values and used a precision of 1×10^−5^.

## Supporting Information

Figure S1 Variable sites in the last repeat of pink VERL. Dots indicate identity with the first row. Each row is an allele, where “p##” denotes the individual and “a” or “b” designates the chromosome. The shaded box highlights the deletion, which clearly separates the 2 clades, as do 5 other SNPs.
(0.05 MB TIF)

Figure S2 Pink VERL repeats show limited divergence in a Southern blot. Two individuals were analyzed, pink35 and pink36. Digestion conditions are as follows: Lane 1- No partial digest. Lane 2- Bpu10I, 30 seconds. Lane 3- Bpu10I, 2 minutes. Lane 4- BsmI, 15 seconds. Lane 5- BsmI, 45 seconds. Lanes “L” are *Hind*III-cut λDNA, and lanes “kb” are a standard ladder with bands ranging from 1.5 to 10 kb. The bottom-most bands (marked “b” or “c”) in the partial digest lanes 2–4 are the fragments where a cut was made in the last repeat. Because the probe was hybridized to the C-terminal end of the array, each band ascending the ladder from “b” or “c” corresponds to a single cut in an interior repeat moving up the array where no other repeat is cut downstream. We did not expect the full ladder of ∼20 repeats because their abundance should decrease with distance from the probe. The only evidence for sequence divergence is where a band is missing (marked “d”) in pink36 lanes 2 and 3. The high molecular weight band (“a”) in lane 1 is the full VERL array from repeats 3 to the last repeat. Note the difference in array size between individuals. DNA was run on a 0.8% agarose gel in TAE before being transferred to a nylon membrane.
(1.38 MB TIF)

Text S1 Text file of PCR primer pairs used in this study.
(0.01 MB RTF)
