# Supplementary figures and images for: Coevolution of Interacting Fertilization Proteins
Source: PLoS Genet. 2009 Jul 24;5(7):e1000570. doi: 10.1371/journal.pgen.1000570 (PMC2704960; doi:10.1371/journal.pgen.1000570)

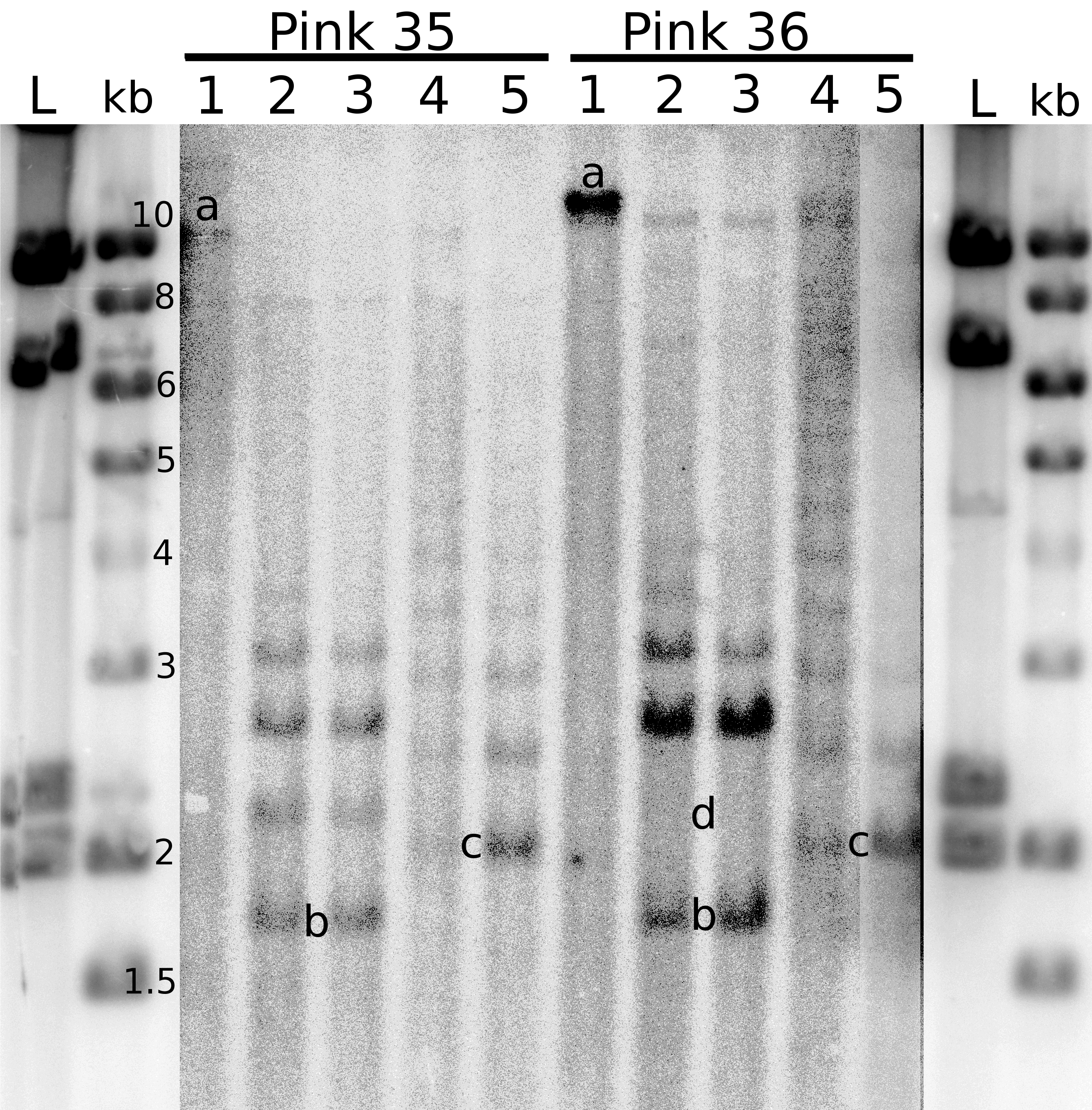

Supplement: Figure S2 — Pink VERL repeats show limited divergence in a Southern blot. Two individuals were analyzed, pink35 and pink36. Digestion conditions are as follows: Lane 1- No partial digest. Lane 2- Bpu10I, 30 seconds. Lane 3- Bpu10I, 2 minutes. Lane 4- BsmI, 15 seconds. Lane 5- BsmI, 45 seconds. Lanes “L” are HindIII-cut λDNA, and lanes “kb” are a standard ladder with bands ranging from 1.5 to 10 kb. The bottom-most bands (marked “b” or “c”) in the partial digest lanes 2–4 are the fragments where a cut was made in the last repeat. Because the probe was hybridized to the C-terminal end of the array, each band ascending the ladder from “b” or “c” corresponds to a single cut in an interior repeat moving up the array where no other repeat is cut downstream. We did not expect the full ladder of ∼20 repeats because their abundance should decrease with distance from the probe. The only evidence for sequence divergence is where a band is missing (marked “d”) in pink36 lanes 2 and 3. The high molecular weight band (“a”) in lane 1 is the full VERL array from repeats 3 to the last repeat. Note the difference in array size between individuals. DNA was run on a 0.8% agarose gel in TAE before being transferred to a nylon membrane. (1.38 MB TIF) [file pgen.1000570.s002.tif]
