# Supplementary material for: Coevolution of Interacting Fertilization Proteins
Source: PLoS Genet. 2009 Jul 24;5(7):e1000570. doi: 10.1371/journal.pgen.1000570 (PMC2704960; doi:10.1371/journal.pgen.1000570)
Supplement: Text S1 — Text file of PCR primer pairs used in this study. (0.01 MB RTF) [file pgen.1000570.s003.rtf]

This file is a list of primer pairs used to amplify gene regions of lysin, VERL, cellulase, hemocyanin, and the non-reproductive proteins described in "Coevolution of Interacting Fertilization Proteins" by Clark, NL et al.  For some pairs information is given about their product size and melting temperatures.  When not indicated primers were designed by Primer3 to have a melting temperature at 60 degrees C.  Primer sets are listed in groups according to the target gene and these genes are separated by a horizontal line.___________________________________________________________________________________________Non-reproductive lociPink EEFTCACCGATACCAGGAAGGACTCCTTCTCCCACTGCTTGTCPink RIBTCGACTGATTGTCCGTTTCATTCGTCAGCCCTACCTTGACPink RTPGCACATTCATCTGGTTGACGACTCTGCGCATGAACATCAGGreen RIBTCGACTGATTGTCCGTTTCATTCGTCAGCCCTACCTTGAC___________________________________________________________________________________________Pink VERL N-terminal repeats  (used as V13&V12  and V13&V14)pV12R  ttggctggaatgctctcpV13F  agccacgacaagagcaaacapV14R  gagcttacaagacgactgatgPink VERL C-terminal repeat(used as pV16&17   and V18&V17   and V16&V19)pV16_CtermRep_F	GCATTCCAGCCAAATTCATCpV17_Cterm_R	GAACGCACATACACTGGGAGTpV18_Cterm_F	CCTGAAGGAAGCGCAACTACpV19_Cterm_R	GTAGTTGCGCTTCCTTCAGG___________________________________________________________________________________________Green VERL N-terminal repeatsused pink VERL N-terminal repeat primers as aboveGreen VERL C-terminal repeatused as pV16&17   and V18&V17   and V16&V19gV18  CCTGAAAGAAGCGCAACCGCgV19  GCGGTTGCGCTTCTTTCAGGgV16 and gV17 match pink exactly (pV16 and pV17 above)___________________________________________________________________________________________Pink Lysin (listed as primer pairs)PLYS0010       gattacaagatgaagctgttggtg           7         24        60.06     PLYS0011       gcgatattaccgagagtgtaacg            559       23        60.06     pcr size: 553	overlap: 0	GC content: 0.39PLYS0020       cgttacactctcggtaatatcgc            537       23        60.06     PLYS0021       gaaatcatcatcggcaaatctaa            1104      23        60.29     pcr size: 568	overlap: 22	GC content: 0.40PLYS0030       ttagatttgccgatgatgatttc            1082      23        60.29     PLYS0031       gtcaactgcagtatttgggtcc             1604      22        60.78     pcr size: 523	overlap: 22	GC content: 0.42PLYS0040       ttgtgcatgcattactgacaaa             1611      22        60.18     PLYS0041       gtttcacagtgtggacgtagctc            2152      23        61.15     pcr size: 542	overlap: -7	GC content: 0.46PLYS0050       cgtccacactgtgaaactgaata            2136      23        60.08     PLYS0051       tgtccagcgagtacaagataagc            2692      23        60.80     pcr size: 557	overlap: 16	GC content: 0.37PLYS0060       aagcttatcttgtactcgctgga            2668      23        59.59     PLYS0061       tttgtggtactgtaaggttaatacgat        3175      27        58.58     pcr size: 508	overlap: 24	GC content: 0.39PLYS0070       tcgtattaaccttacagtaccacaaa         3150      26        58.29     PLYS0071       catttgacttacattacacgcga            3718      23        60.07     pcr size: 569	overlap: 25	GC content: 0.35PLYS0080       tcgcgtgtaatgtaagtcaaatg            3696      23        60.07     PLYS0081       actcttgtgtgagagatgaagcc            4208      23        59.94     pcr size: 513	overlap: 22	GC content: 0.36PLYS0090       ggcttcatctctcacacaagagt            4186      23        59.94     PLYS0091       tgttgttctcatcttctgacagc            4736      23        59.53     pcr size: 551	overlap: 22	GC content: 0.37PLYS0100       tgctgtcagaagatgagaacaac            4713      23        59.53     PLYS0101       taaccgactgtagtcagcaacaa            5219      23        59.86     pcr size: 507	overlap: 23	GC content: 0.38PLYS0110       gacctccagttgttgctgactac            5188      23        60.22     PLYS0111       atgggttgctgaagacctattct            5720      23        60.37     pcr size: 533	overlap: 31	GC content: 0.40PLYS0120       atccaggcagaataggtcttca             5690      22        60.10     PLYS0121       ccttctactgcaagtatcggcta            6197      23        59.95     pcr size: 508	overlap: 30	GC content: 0.43PLYS0130       tagccgatacttgcagtagaagg            6175      23        59.95     PLYS0131       caacaaatagccaacccatgtt             6665      22        60.98     pcr size: 491	overlap: 22	GC content: 0.44>patch1 plys1.5CGATCTGAAACCCTGCAGTTGTGCCGCCGAATACACTTAC>patch2	plys4.5GGCGTAAAACAACCAACCAACTTTCCGCCATGTGTGTATG>patch3	plys6.5AATGTGCGTCGATTACGAGAGCCGCAGGCTATAAACATTA>patch4	plys7.5GGCGCATTTTACAGCAAGAGATCGTGTTCAAGCCGAGAAT>patch5	plys8.5TTTCGCCAACTTCTGAGCTTTTAAAGCCCCACCCTGACTA>patch6	plys9.5AAACACTTCGGGTTGACTGGTTCCAACAAACCACCATAGG>patch7	plys9.7CCTTTTGTAAATGGCCGAATCTGCCTGGTAGGTTCTACGC___________________________________________________________________________________________Green Lysinglys ex1_F ex2_ROLIGO            start  len      tm     gc%   any    3'   rep seq LEFT PRIMER         22   20   60.38   45.00  2.00  0.00 12.00 GTTGGTGCTTTGGGTTTTTGRIGHT PRIMER       271   21   59.85   52.38  6.00  3.00  9.00 CGGTCAGCTGTCTATCGAATCglys ex2_F ex3_ROLIGO            start  len      tm     gc%   any    3'   rep seq LEFT PRIMER         40   20   61.23   55.00  6.00  2.00 12.00 ATAGACAGCTGACCGCATGGRIGHT PRIMER       289   20   59.17   50.00  4.00  2.00 10.00 TGGGTCTGCATGTAACGTCTglys ex3_F ex4_ROLIGO            start  len      tm     gc%   any    3'   rep seq LEFT PRIMER         38   20   60.18   55.00  4.00  3.00 11.00 CGTTACATGCAGACCCACTGRIGHT PRIMER       283   21   60.76   57.14  3.00  2.00  9.00 CCGAGTGTAGTCACCAACAGCglys ex4_F ex5_ROLIGO            start  len      tm     gc%   any    3'   rep seq LEFT PRIMER         92   20   59.73   45.00  4.00  1.00 11.00 GGAGAAAAATGATCCCACCARIGHT PRIMER       329   20   59.83   45.00  4.00  0.00  9.00 TTCCCGTGGTTTTTAACTGG___________________________________________________________________Haemocyanin from 8 species>Capture remainder of Exon12OLIGO            start  len      tm     gc%   any    3' seq LEFT PRIMER         22   19   60.67   52.63  4.00  1.00 ATGGCGACGTTTCCTCACTRIGHT PRIMER       999   20   60.16   45.00  2.00  0.00 ATTCACAGCAACAACCGACAPRODUCT SIZE: 978, PAIR ANY COMPL: 3.00, PAIR 3' COMPL: 1.00>ex12_F2 & R2  New primers based on species specific information and codon positions.OLIGO            start  len      tm     gc%   any    3' seq LEFT PRIMER          1   20   60.51   55.00  2.00  0.00 ctgtgccttactgggattgg  <--based on black and 3'anchored at W(TGG)RIGHT PRIMER       192   20   58.85   50.00  3.00  0.00 TGAAGAAGTGACGGTGGAAG    <--same in red,green,flat,pink,blackPRODUCT SIZE: 927bp, PAIR ANY COMPL: 3.00, PAIR 3' COMPL: 0.00>Primers from K. Streit, Geiger, Lieb / Molecular phylogeny and the geographic origin of haliotidae traced by haemocyanin sequences.Used to amplify white and pinto.HtH1e:  GTTTTCCATGCTGATGAACHtH1g rev: GTGGCCATCAGGCATGGG>ex12_13_F2  and R2   improvements over tuberculata primers with species specific sequence from red and flat and codon positions. 2 LEFT PRIMER          4   19   60.12   57.89  4.00  0.00 cggtgGTAGCTGTGAATGG   RIGHT PRIMER       199   21   60.03   38.10  5.00  3.00 AATTGCTTGAAAACCGTTGTG   PRODUCT SIZE: 620, PAIR ANY COMPL: 4.00, PAIR 3' COMPL: 0.00___________________________________________________________________Cellulase>Exons 2&3OLIGO            start  len      tm     gc%   any    3' seq LEFT PRIMER          8   20   59.83   50.00  3.00  1.00 CACAGCCAATGTCCAGAAGARIGHT PRIMER      1179   20   60.27   55.00  4.00  2.00 GAGTCACCTCGCCACTTGATPRODUCT SIZE: 1172, PAIR ANY COMPL: 4.00, PAIR 3' COMPL: 3.00>alternate reverse cel23_R2RIGHT PRIMER       196   20   59.65   55.00  3.00  1.00 ccatcataccaccctccagt>Exons 3&4OLIGO            start  len      tm     gc%   any    3' seq LEFT PRIMER          1   20   57.88   50.00  4.00  2.00 AGGAGGCGGTACAGAAAGATRIGHT PRIMER      1190   20   59.37   45.00  7.00  3.00 TGGAATCCAGCACTTCAAGAPRODUCT SIZE: 1190, PAIR ANY COMPL: 3.00, PAIR 3' COMPL: 1.00>Exons 4,5,&6OLIGO            start  len      tm     gc%   any    3' seq LEFT PRIMER          1   20   60.78   55.00  7.00  3.00 AGCTGGTGACCACGTCAAGTRIGHT PRIMER       779   20   60.42   55.00  4.00  2.00 CGAGTAGAACTGCCCAGCATPRODUCT SIZE: 779, PAIR ANY COMPL: 5.00, PAIR 3' COMPL: 2.00>Exons6&7OLIGO            start  len      tm     gc%   any    3' seq LEFT PRIMER         16   20   59.75   50.00  4.00  2.00 CGCCACACTTCTGTCAACATRIGHT PRIMER       993   20   60.22   55.00  4.00  2.00 TTGTCGTCCCATCCTAGAGCPRODUCT SIZE: 978, PAIR ANY COMPL: 3.00, PAIR 3' COMPL: 0.00>Exons7&8OLIGO            start  len      tm     gc%   any    3' seq LEFT PRIMER          9   23   60.08   43.48  3.00  0.00 TGGTTACAAAGACGAGATGTGTGRIGHT PRIMER       711   20   59.55   50.00  6.00  0.00 TATCTGTTGGAGCCCCACTTPRODUCT SIZE: 703, PAIR ANY COMPL: 5.00, PAIR 3' COMPL: 3.00RED-specific primers>Exon4_R red-specific  to be used with cel34_FRIGHT PRIMER       194   21   57.07   42.86  2.00  2.00 TCTGTCTGTTTCATCGTAGCA>Exon4F2 and 4R2 red-specific  (R2 can be used with cel34_F)OLIGO            start  len      tm     gc%   any    3' seq LEFT PRIMER         28   20   60.37   50.00  5.00  2.00 CAGGGTAAATTCCGTGTTGC  located just before exon3RIGHT PRIMER       273   20   60.62   50.00  6.00  0.00 ATGGGATCCAGCACTTGAGA located w/in exon4 near endPRODUCT SIZE: 1210, PAIR ANY COMPL: 4.00, PAIR 3' COMPL: 1.00GREEN-specific primers>Exon5_FLEFT PRIMER          3   20   61.77   55.00  4.00  0.00 cgctgaggacatgaagatggTo be used with cel456_R. This primer also matches discus perfectly.  Its last 4to2 nt are based on a Methionine codon (ATG) and the last is a first codon position.>Exon5_RRIGHT PRIMER       194   20   59.29   60.00  5.00  3.00 ctagagaggcagcgatctccTo be used with cel456_F. Contains only 1 mismatch with discus at nt 7th from end.PINK-specific primers>Exon5_FLEFT PRIMER          2   21   58.97   42.86  3.00  2.00 ggttggagaaggaaatgatgaTo be used with cel456_R.  Also matches discus perfectly.>Exon5_RRIGHT PRIMER       231   20   59.66   55.00  3.00  0.00 GCTATCTCCCCAGCAACATCTo be used with cel456_F.  One mismatch with discus 3rd from 5' end. >Exon4_RTo be used with cel34_FRIGHT PRIMER       197   20   60.62   50.00  6.00  0.00 atgggatccagcacttgagaRIGHT PRIMER       191   20   60.62   55.00  4.00  2.00 CATAGGCATCCTTCCACTGC pre&postExon2OLIGO            start  len      tm     gc%   any    3' seq LEFT PRIMER          1   20   59.83   50.00  3.00  1.00 CACAGCCAATGTCCAGAAGARIGHT PRIMER       200   17   59.94   64.71  4.00  2.00 acCTGTGTGGGGCACCT 	~230 basepairspre&postExon3OLIGO            start  len      tm     gc%   any    3' seq LEFT PRIMER          1   20   60.37   50.00  5.00  2.00 CAGGGTAAATTCCGTGTTGC RIGHT PRIMER       264   20   60.14   55.00  5.00  0.00 CAGTCAGCGGGTGATATGTG 	PRODUCT SIZE: 290, PAIR ANY COMPL: 3.00, PAIR 3' COMPL: 1.00pre&postExon4OLIGO            start  len      tm     gc%   any    3' seq LEFT PRIMER         68   21   60.12   47.62  4.00  3.00 TTATCCCTCGGGTATCGAATC 	 RIGHT PRIMER       357   20   60.11   45.00  4.00  2.00 CTTGTGTCGCCATGATTTTG 	  PRODUCT SIZE: ~340, PAIR ANY COMPL: 4.00, PAIR 3' COMPL: 2.00preExon5OLIGO            start  len      tm     gc%   any    3' seq LEFT PRIMER         44   20   59.55   50.00  8.00  2.00 ATCAAGTGGCCCTTGGACTA    RIGHT PRIMER       278   20   59.66   55.00  3.00  0.00 GCTATCTCCCCAGCAACATC  
